# Risk factors for oesophageal, lung, oral and laryngeal cancers in black South Africans

**DOI:** 10.1038/sj.bjc.6600338

**Published:** 2002-06-05

**Authors:** R Pacella-Norman, M I Urban, F Sitas, H Carrara, R Sur, M Hale, P Ruff, M Patel, R Newton, D Bull, V Beral

**Affiliations:** MRC/CANSA/NHLS/WITS Cancer Epidemiology Research Group, National Cancer Registry, PO Box 1038, Johannesburg 2000, South Africa; Department of Anatomical Pathology, National Health Laboratory Services (formerly South African Institute for Medical Research) and University of the Witwatersrand, York Road, Parktown 2196, South Africa; Department of Radiation Therapy, University of the Witwatersrand, York Road, Parktown 2196, South Africa; Department of Medical Oncology, University of the Witwatersrand, York Road, Parktown 2196, South Africa; Department of Haematology, University of the Witwatersrand, York Road, Parktown 2196, South Africa; Cancer Research UK, Epidemiology Unit, Oxford, UK

**Keywords:** epidemiology, case–control, oesophagus, lung, oral, larynx

## Abstract

The authors used data collected from 1995 to 1999, from an on-going cancer case–control study in greater Johannesburg, to estimate the importance of tobacco and alcohol consumption and other suspected risk factors with respect to cancer of the oesophagus (267 men and 138 women), lung (105 men and 41 women), oral cavity (87 men and 37 women), and larynx (51 men). Cancers not associated with tobacco or alcohol consumption were used as controls (804 men and 1370 women). Tobacco smoking was found to be the major risk factor for all of these cancers with odds ratios ranging from 2.6 (95% CI 1.5–4.5) for oesophageal cancer in female ex-smokers to 50.9 (95% CI 12.6–204.6) for lung cancer in women, and 23.9 (95% CI 9.5–60.3) for lung cancer and 23.6 (95% CI 4.6–121.2) for laryngeal cancer in men who smoked 15 or more grams of tobacco a day. This is the first time an association between smoking and oral and laryngeal cancers has been shown in sub-Saharan Africa. Long-term residence in the Transkei region in the southeast of the country continues to be a risk factor for oesophageal cancer, especially in women (odds ratio=14.7, 95% CI 4.7–46.0), possibly due to nutritional factors. There was a slight increase in lung cancer (odds ratio=2.9, 95% CI 1.1–7.5) in men working in ‘potentially noxious’ industries. ‘Frequent’ alcohol consumption, on its own, caused a marginally elevated risk for oesophageal cancer (odds ratio=1.7, 95% CI 1.0–2.9, for women and odds ratio=1.8, 95% CI 1.2–2.8, for men). The risks for oesophageal cancer in relation to alcohol consumption increased significantly in male and female smokers (odds ratio=4.7, 95% CI=2.8–7.9 in males and odds ratio=4.8, 95% CI 3.2–6.1 in females). The above results are broadly in line with international findings.

*British Journal of Cancer* (2002) **86**, 1751–1756. doi:10.1038/sj.bjc.6600338 www.bjcancer.com

© 2002 Cancer Research UK

## 

South Africa is a society in transition, particularly for the black population. Johannesburg and its surrounding areas in Gauteng province, including the ‘dormitory’ townships of Soweto, form the major economic powerhouse of the country. As such the area attracts migrants from all areas of the country as well as from other African (and also non-African) countries. The population of Gauteng province reached 7.3 million in 1996 of which 97% were urban residents (Statistics South Africa, 1998).

In addition to urbanisation, recent decades have seen many lifestyle changes for black Africans, including increasing rates of smoking and alcohol consumption (Yach and Townsend, 1988; Segal *et al*, 1988; Strebel *et al*, 1989), both known risk factors for oesophageal, lung, oral and laryngeal cancers among others (IARC, 1986, 1988; Doll *et al*, 1994). Since the repeal of prohibitive alcohol legislation for blacks in 1961 there has been a steady shift among black drinkers from the consumption of traditional alcoholic beverages to Western style drinks (Segal *et al*, 1988).

The most recent estimated combined lifetime risk for developing oesophageal, lung, oral, laryngeal, or naso-pharyngeal cancer was 1 in 20 for black South African males and 1 in 76 for black South African females (Sitas *et al*, 1998). Indications of a role for smoking in the development of lung cancer among more urbanised African men from Johannesburg and Natal in South Africa, and Bulawayo in Zimbabwe, have been observed since the 1960s with the incidence of lung cancer already beginning to rise at that time (Bradshaw and Schonland, 1969; Parkin *et al*, 1994). More recently, a case–control study from the Northern Province of South Africa (which is 88% rural) has described an association between smoking and lung cancer for African men and, for the first time, for African women (Mzileni *et al*, 1999).

Previous case–control studies from South Africa (Bradshaw and Schonland, 1969, 1974; van Rensburg *et al*, 1985; Sammon, 1992) and Zimbabwe (Vizcaino *et al*, 1995) showed an elevated risk for the development of oesophageal cancer with tobacco smoking; and various international (Tuyns *et al*, 1979; Day, 1984) and local (McGlashan *et al*, 1982) studies, the most recent by Segal *et al* (1988), have suggested that alcohol and tobacco consumption have independent and combined risk effects for this cancer. The incidence of oesophageal cancer has been increasing in South Africa since the 1950s (Segal *et al*, 1988), with the risk being much higher than the national average for those living in the Eastern Cape Province, particularly the rural areas of the former Transkei ‘homeland’ (Rose, 1973; Makaula *et al*, 1996) where nutritional factors and consumption of *Fusarium sp* contaminated maize may be important contributing factors (see Gelderblom *et al*, 1988).

There have been no sub-Saharan African studies looking at the relative importance of tobacco and/or alcohol in the development of cancers of the mouth, pharynx and larynx.

An ongoing cancer epidemiological study began in 1995 in the three main public referral hospitals of greater Johannesburg to collect data on the importance of selected potential risk factors for cancer. The present analysis aims to provide recent estimates of the relative importance of some of the suspected risk factors for oesophageal, lung, oral and laryngeal cancers in these patients: place of birth/residence, education, exposure to domestic or industrial pollutants, tobacco use, and alcohol consumption.

## METHODS

### Study design

Nurses, trained in interviewing, questioned adult African patients (black patients all of whose ancestors came from the African continent) with newly diagnosed cancers at Chris Hani Baragwanath, Hillbrow, and Johannesburg Hospitals using a structured two page questionnaire (available on request). The interview was conducted in the preferred language of the patient (usually Zulu or seSotho) following written or verbal (if illiterate) consent to participate. The questionnaires were anonymous and included questions on smoking, frequency of alcohol consumption, birthplace, residence, education, and reproductive, contraceptive and lifetime sexual history. For 90% of patients the diagnosis was confirmed by histology, haematology, or cytology. Cancers were classified by primary site and morphology using the WHO International Classification of Diseases for Oncology (ICD 0-2) guidelines (WHO, 1990). The study was approved by the University of the Witwatersrand ethics committee.

This analysis of oesophageal, lung, oral, and laryngeal cancers included 1586 females and 1314 males who were interviewed between March 1995 and April 1999. A total of 1348 patients, who had cancers thought to be associated with the effects of tobacco and/or alcohol, were excluded i.e. cancers of the stomach, bladder, liver, pancreas, nasopharynx, and uterine cervix plus cancer of the larynx in women (eight cases, among whom two were current and two ex-smokers). Male cases included oesophageal (275), lung (105), oral (87) and laryngeal (51) cancers, whereas female cases included oesphageal (138), lung (41) and oral (37) cancers. The control group of 1370 female patients and 804 male patients consisted of the following cancers: breast (609 females and 15 males); prostate 235; leukaemias (122 females and 109 males); lymphomas (86 females and 115 males); myelomas (50 females and 61 males); ovary 88; endometrium 94; vulva 55; Kaposi's sarcoma (42 females and 73 males); other skin (14 female and 13 male); colon (68 female and 61 male); penis 14; and other malignancies (142 female and 108 males).

### Risk categories

Patients were grouped according to province of birth: Gauteng province, which includes Johannesburg and Soweto, was used as the reference category; Eastern Cape Province was singled out as it is a known high risk area for oesophageal cancer (Makaula *et al*, 1996); the remaining seven provinces; and foreign.

The South African Central Statistical Services' Standard Occupational Classification manual (Central Statistical Services, 1986) was used to classify patients according to the type of industry/workplace in which they usually worked. The reference category included managerial, administrative, clerical and sales personnel as well as those not economically active (housewives, students, and the long term unemployed). The group called ‘potentially noxious’ industries included: metal and non-metallic mineral, chemical, petroleum, coal, rubber, plastics, wood and paper manufacturing and processing; the motor vehicle industry; construction; and mining and quarrying.

To take into account the possibility that some patients may have given up smoking due to their illness, those who stopped smoking more than 5 years prior to the date of interview were classified as ex-smokers while those who smoked within 5 years of the date of interview were classified as current smokers. Current smokers were then subdivided into ‘light’ (1–14 g day^−1^) and ‘heavy’ (>=15 g day^−1^) current smokers, assuming weights of 1 g for commercial cigarettes; 1 g for hand rolled cigarettes; and a conservative 1 g pipe^−1^.

The frequency of consumption of alcohol from maize, from sorghum, and from other traditional home-brewed beers, commercial beer, wine, commercial and home-distilled spirits, and other alcoholic drinks was recorded as ‘never’, ‘less than once a week’, ‘more than once a week’, and ‘most days’. Patients who drank any alcoholic beverage less than once a week were called ‘occasional drinkers’; those who took any alcoholic drink one to three times a week ‘weekly drinkers’; and those who reported consuming at least one type of alcoholic beverage on most days ‘frequent drinkers’.

### Statistical analyses

Relative risks associated with each risk factor for the cancers studied were estimated by using the odds ratios derived from unconditional, unmatched multiple logistic regression (SAS, 1988). A separate term was used for each adjustment factor in the model. A second model was then built by removing exposures that were not statistically significant at the 5% level. As this made virtually no difference to the results, only the fully adjusted model is presented.

## RESULTS

The distribution of cases and controls according to place of birth, education, heating fuel, work environment, tobacco use, and alcohol consumption is shown in Tables 1

**Table 1:**
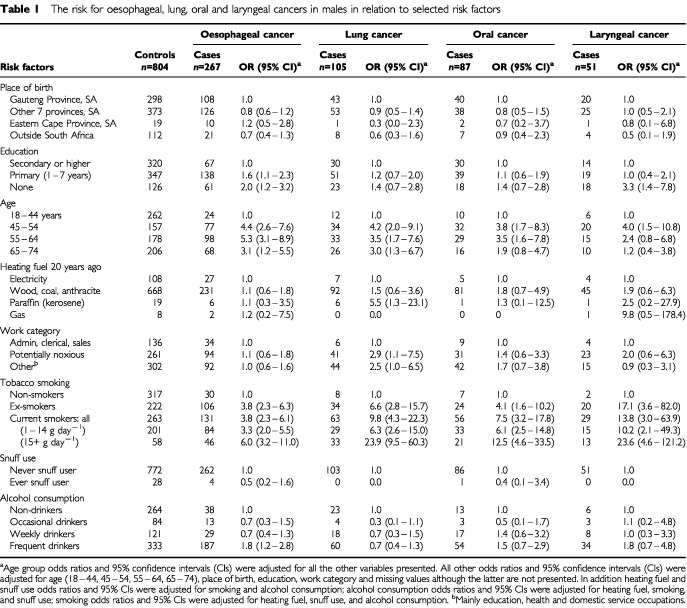
The risk for oesophageal, lung, oral and laryngeal cancers in males in relation to selected risk factors

and 2

**Table 2:**
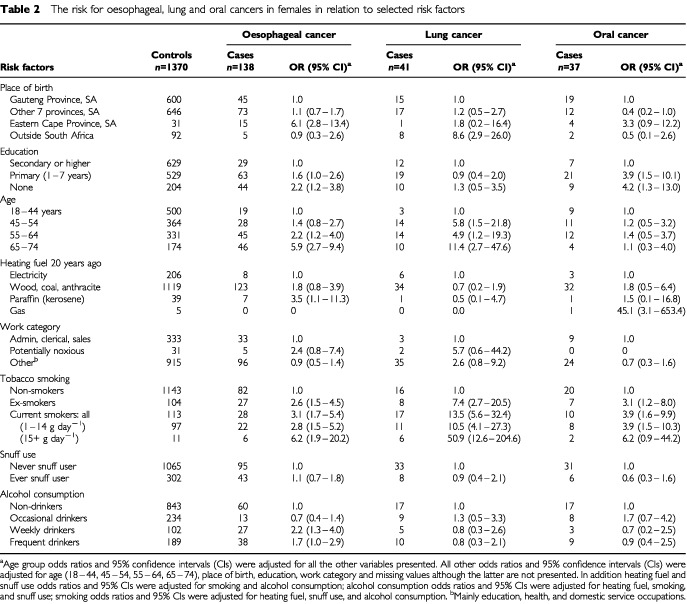
The risk for oesophageal, lung and oral cancers in females in relation to selected risk factors

.

Women who were born in the Eastern Cape Province had a significantly increased risk [Odds Ratio (OR)=6.1] of being diagnosed with oesophageal cancer compared to those born elsewhere in South Africa or outside of the country. When duration of residence in the Eastern Cape was looked at (Table 3

**Table 3:**
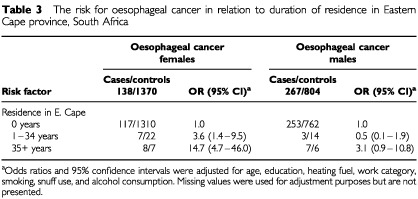
The risk for oesophageal cancer in relation to duration of residence in Eastern Cape province, South Africa

) length of residence had a large effect for women (OR=3.6 for 1–34 years; OR=14.7 for 35 or more years) but was only marginally significant for men who had lived there for 35 or more years (OR=3.1).

Compared with eight or more years of education, lower levels were associated with elevated odds ratio estimates for oesophageal cancer of 2.0 for men with no education, 1.6 for men with only primary education, and 2.2 for women with no education. Oral cancers were about four times more frequent in women with no or only primary education (OR=4.2 and 3.9), compared to those with secondary or higher education; this difference was not found for these cancers in men. However males with no education were more likely to be diagnosed with laryngeal cancer (OR=3.3).

Using ages 18–44 as the base line, in women there was a clear trend for increasing risk of oesophageal cancer with increasing age; the trend was less clear for men where 55–64 year olds have the greatest estimated risk. With respect to lung cancer, women 65–74 had approximately twice (OR=11.4) the estimated risk compared to those aged 45–64. This was in contrast to men whose risk was three to four times base line at all ages 45 and over. Age was not a risk factor for oral cancer in women and possibly not for laryngeal cancer in men (only showing a significant increase in men aged 45–54); however it was significantly associated with oral cancers in men 45–64 years old.

At the time of their interview 73% of controls reported using electricity for both cooking and heating, with only 18% using wood, charcoal, coal, or anthracite. In the past the situation was quite different with 82% reporting the use of the latter fuels, and only 15% using electricity ‘20 years ago’.

The reported use of wood, charcoal, coal or anthracite for heating ‘20 years ago’ was not associated with any significant increase in the cancers studied. However, although the numbers were small, men, but not women, who reported using liquid paraffin (kerosene) for heating were at greater risk for lung cancer (OR=5.5) and women, but not men, using this fuel were at increased risk for oesophageal cancer (OR=3.5).

Using broad groupings for industrial/workplace classifications, males working in areas with ‘potentially noxious’ exposures had increased risks: they were 2.9 times more likely to be diagnosed with lung cancer than their counterparts in administrative, clerical and sales businesses. There were too few women (n=38) working in ‘potentially noxious’ environments to draw inferences.

Tobacco smoking, past or current, was the major risk factor for all of the cancers included in this study. For cases the mean duration of smoking was 22.1 (s.d.±19.0) years for males and 6.4 (s.d.±14.3) years for females, compared to 3.5 (s.d.±9.8) and 0.8 (s.d.±5.0) years respectively for controls.

Ex-smokers and ‘light’ current smokers had about a three-fold increased risk for oesophageal cancer, being slightly higher for men than for women. For ‘heavy’ smokers the risk doubled to an OR of about six in both sexes.

Lung cancer was six to 13 times more likely to be diagnosed in ex- or ‘light’ smokers, with the risk being slightly higher in women than in men. In the case of ‘heavy’ smokers the odds ratios were 23.9 in men and 50.9 in women, both with wide confidence limits.

The increased risk of oral cancers in women who smoked was similar to that for oesophageal cancers; however, for men it was somewhat increased to an OR of 12.5 in ‘heavy’ smokers.

Almost all of the men (49/51) with laryngeal cancer had smoked at some time, with increased risks ranging from an OR of 10.2 for ‘light’ smokers to an OR of 23.6 for ‘heavy’ smokers.

Snuff use was more common among women than smoking, with 22% of female control patients and 4% of males reporting this habit. ‘Ever’ use of snuff as compared to ‘never’ use did not appear to be associated with an increased risk of oesophageal, lung, or oral cancer.

Frequency of alcohol consumption, on its own, was not a major contributor to the cancers studied. The only significant increased risk was for oesophageal cancer in women classified as ‘weekly drinkers’ (OR=2.2) and women (OR=1.7) and men (OR=1.8) classified as ‘frequent drinkers’. In addition, when alcohol consumption was combined with smoking the risk of the association with oesophageal cancer was significantly increased (OR=4.4 men and women combined; see Table 4

**Table 4:**
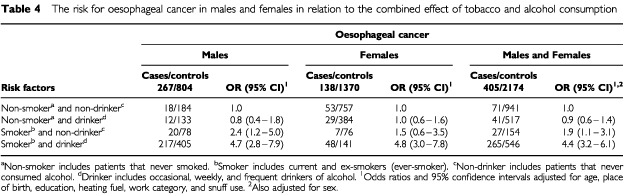
The risk for oesophageal cancer in males and females in relation to the combined effect of tobacco and alcohol consumption

).

## DISCUSSION

The findings of this analysis of oesophageal, lung, oral and laryngeal cancers diagnosed in black patients at state hospitals in Johannesburg are broadly in line with international findings, including studies on lung cancer in Zimbabwe (Parkin *et al*, 1994), and Northern Province, South Africa (Mzileni *et al*, 1999); and on oesophageal cancer in Zimbabwe (Parkin *et al*, 1994) and in Soweto (Segal *et al*, 1988).

Tobacco smoking was the leading risk factor for all of the cancers analysed with both current and former use harmful, and the expected dose-response effect seen. This is the first time an association between smoking and oral and laryngeal cancers has been shown in sub-Saharan Africa. Estimates for smoking among South Africans vary considerably. In addition there is a divergence of usage between different communities, with smoking among mixed race men and women being equally common, smoking by black women much lower than by black men, and with the white community having intermediate rates (Reddy *et al*, 1996). The current cigarette smoking rates in our control group were 32.8% for men and 8.3% for women. This compares to rates of 55% and 10% respectively obtained for black adults in Gauteng from a multi-stage cluster sample survey conducted in February 1995 (Reddy *et al*, 1996). The lower rates found in our study could partially be explained by the fact that our study does not cover individuals who have access to private or company medical care.

The much lower smoking rates among our female cases (45.4%) when compared to our male cases (90.8%) indicates other causes for the cancers studied in addition to those examined. These could include *inter alia* passive smoking (in the 1995 survey (Reddy *et al*, 1996) 48% of all adults reported exposure to smoking by at least one household member), the presence of various infectious and inflammatory conditions, genetic, hormonal and nutritional (males traditionally receiving the best and the most of food when there are shortages) factors.

Figures from the (SA) National Council Against Smoking (Saloojee, 2000) indicate that legal sales of commercial cigarettes have fallen every year since peaking at 40 billion in 1990; the figure for 1998 stood at 30 billion. Between 1994 and 1999 real excise taxes on cigarettes went up by 149%. Recent years have also seen the introduction of tobacco control legislation including severe restrictions on advertising and the banning of smoking in public places. It can therefore be optimistically expected that South Africa is past the peak of its tobacco consumption epidemic and that tobacco related cancers will begin to decline in time. In contrast little effort has been made to curb alcohol abuse. Total adult per capita pure alcohol consumption was estimated to be 10 l in 1995 and had increased by over 50% since 1970 (WHO, 1999).

A more in depth analysis of oesophageal cancer was carried out because of its known high occurrence (e.g. 35 and 18 per 100 000 in ‘Bantu’ (black) men and women respectively in 1955–1969 (Rose, 1973)) in the Transkei region of southeastern South Africa where a number of studies have been conducted over the last half-century. Our data confirm a higher risk, following adjustment for other factors, for those patients who had lived in that area. Contrary to studies conducted in the Transkei itself we found greater risks for women (14.7) than for men (3.1) with 35 or more years of residence. This can be explained by the fact that the Transkei studies looked at individuals who were probably full-time residents of that area whereas in our study especially the men may well have been migrant workers who still considered Transkei as their home but who worked in the Johannesburg area for much of the time.

Many of the world's ‘hot spots’ for oesophageal cancer are in populations who are poor and who consume restricted diets. Maize, which has low levels of niacin, riboflavin, vitamin C and other micro-nutrients, is the staple in the Transkei area. In addition home-grown and stored maize is often contaminated with *Fusarium* species which produce mycotoxins recognised as being ‘possibly carcinogenic’ by an IARC (International Agency for Research on Cancer) working group (IARC, 1993). Contaminated ears are often used for brewing beer while the ‘good’ ears are consumed as porridge.

The fact that less years of formal schooling was associated with increased risk for oesophageal cancer in both men and women could be due to nutritional deficiencies as a result of ignorance or of lower income.

Worldwide, numerous case–control and cohort studies have shown that both tobacco and alcohol increase the risk of oesophageal cancer, and that their joint effect is multiplicative (IARC, 1986, 1988). In contrast to several southern African studies from the 1960s and 1970s which failed to show this effect (e.g. Bradshaw and Schonland, 1974; Vizcaino *et al*, 1995), the current study agrees with the majority. Many earlier studies were conducted in areas where, and at times when, the principal beverages had a low (2–4%) alcohol content possibly giving increased risks that were undetectable.

Contrary to what might be expected, use of wood, coal or anthracite for heating was not a significant risk factor for any of the cancers presented here. This is probably because cumulative exposure was low since heating in the Johannesburg area is only needed at night for about 3 months of the year, winter days generally being sunny and mild. In contrast, inexplicably, use of liquid paraffin was a significant risk factor for lung cancer in men (only one woman with lung cancer used paraffin) and for oesophageal cancer in women.

Although not statistically significant, probably because of very small numbers in the reference category, use of fossil fuels for heating in the past, and employment in ‘potentially noxious’ industries (for men) did lead to increased risks for oral cancers. This is in accordance with known risk factors for these cancers (Johnson, 1999). We were unable to detect significant risks for alcohol use and oral cancers since we did not have information on quantity – oral cancers increasing in individuals consuming 21 or more drinks a week (Franceschi *et al*, 1999). We also did not detect any effect of snuff use but we did not separate oral and inhaled snuff, the latter being much more common in our communities. The risks for laryngeal cancers were very similar to those for the oral cancers.

In summary, the well known risk factors for oesophageal, lung, oral, and laryngeal cancers hold for the black population of greater Johannesburg.
